# Disulfide HMGB1 acts via TLR2/4 receptors to reduce the numbers of oligodendrocyte progenitor cells after traumatic injury in vitro

**DOI:** 10.1038/s41598-021-84932-0

**Published:** 2021-03-17

**Authors:** R. Ved, F. Sharouf, B. Harari, M. Muzaffar, S. Manivannan, C. Ormonde, W. P. Gray, M. Zaben

**Affiliations:** 1 Neuroscience and Mental Health Research Institute, Haydn Ellis Building, Cathays, Cardiff, CF24 4HQ UK; 2 Division of Psychological Medicine and Clinical Neurosciences (DPMCN), School of Medicine, Cardiff University, Cardiff, CF24 4HQ UK

**Keywords:** Cellular neuroscience, Molecular neuroscience, Neuroimmunology

## Abstract

Traumatic brain injury (TBI) is associated with poor clinical outcomes; autopsy studies of TBI victims demonstrate significant oligodendrocyte progenitor cell (OPC) death post TBI; an observation, which may explain the lack of meaningful repair of injured axons. Whilst high-mobility group box-1 (HMGB1) and its key receptors TLR2/4 are identified as key initiators of neuroinflammation post-TBI, they have been identified as attractive targets for development of novel therapeutic approaches to improve post-TBI clinical outcomes. In this report we establish unequivocal evidence that HMGB1 released in vitro impairs OPC response to mechanical injury; an effect that is pharmacologically reversible. We show that needle scratch injury hyper-acutely induced microglial HMGB1 nucleus-to-cytoplasm translocation and subsequent release into culture medium. Application of injury-conditioned media resulted in significant decreases in OPC number through anti-proliferative effects. This effect was reversed by co-treatment with the TLR2/4 receptor antagonist BoxA. Furthermore, whilst injury conditioned medium drove OPCs towards an activated reactive morphology, this was also abolished after BoxA co-treatment. We conclude that HMGB1, through TLR2/4 dependant mechanisms, may be detrimental to OPC proliferation following injury in vitro, negatively affecting the potential for restoring a mature oligodendrocyte population, and subsequent axonal remyelination. Further study is required to assess how HMGB1-TLR signalling influences OPC maturation and myelination capacity.

## Introduction

Primary brain injury is the term used to describe injury to brain tissue due to sheer forces at the time of TBI. Secondary brain injury is a term coined to describe ongoing tissue damage in the hours-days-week (or possibly even years) after the traumatic incident. The larger therapeutic window and slower disease processes make modulation of secondary brain an attractive strategy in the search for therapeutic options for TBI patients. Therefore, development of a deeper mechanistic understanding of secondary brain injury is vital.

Post-traumatic secondary brain injury is a process that follows the physical injury, which may be amenable to modulation^1–3^. Since the pathophysiology implicated in secondary brain injury is not yet fully understood, therapeutic strategies targeting this phase of brain injury are yet to be successfully implemented in clinical practice^3,4^. Neuroinflammation has been implicated as a key feature of secondary brain injury, as evidenced through the upregulation of numerous proinflammatory cytokines following TBI such as TNF-alpha, IL-1 and IL 6, all of which appear to be detrimental to recovery^5^. The slower temporal development of neuroinflammatory secondary brain injury has prompted study of modulation of the neuroinflammasome as a potential route to mitigate the detrimental impact of TBI.

Recent studies have identified high mobility group box-1 (HMGB1), as a protein that propagates inflammation following TBI^3,6^. It is one of the ‘damage associated molecular pattern’ (DAMP) molecules, which stimulate the release of the pro- neuroinflammatory factors described above^6^. Following TBI, HMGB1 is passively released from injured and necrotic cells^7^. The release of HMGB1 from necrotic cells serves as an endogenous “danger signal” that primes immune system to the presence of the injury and increases the activation of neutrophils, monocytes, cytokines, and natural killer cells, and activates microglia, thereby propagating inflammation^3^. Once released into the extracellular milieu, HMGB1 binds to the transmembrane toll-like receptors (TLR) 2 and 4, and the receptor for advanced glycation end products (RAGE)^8,9^. Therefore, these pathways may be mediators of brain damage after TBI, and could potentially be amenable to therapeutic modulation.

Injury to white matter, including the myelinating cells, the oligodendrocytes, is also now more appreciated as a cause of many of the symptoms TBI patients may develop, such a cognitive issues and psychomotor disability^10^. In normal white matter, a single oligodendrocyte maintains myelin sheaths around a numerous axons^11^. Sheer forces after a TBI cause traumatic axonal injury (TAI), whereby the myelin sheath around axons becomes disrupted, impairing subsequent neuronal communication^10^. Recovery following TAI within the brain is dependent on numerous factors, including the severity of injury and the ability of axons to become remyelinated^12^. This may rely on new oligodendrocytes being generated, migrating to the site of injury, and subsequent maturation to remyelinate the injured axons^13,14^. This has led to interest in the study of the oligodendrocyte progenitor cells, (OPC). These cells comprise up to 5% of the cell population within the adult brain^15^. They express the chondroitin sulphate Neurone Glia 2 (NG2). This population of cells has been shown to have the ability to mature into fully formed oligodendrocytes in vitro and in vivo^16^. NG2^+^ OPCs are therefore purported to be a potential source of remyelinating cells following TBI^17–19^.

Elucidating whether these OPCs are influenced by the neuroinflammatory mediators, such as HMGB1, released after TBI could be a step towards identifying a pathway which could be a translational target for the development of novel therapeutic agents to improve clinical outcomes after neurotrauma^6^. The aim of the present study was to identify the impact of the HMGB1 upon NG2^+^ cells in culture, incorporating study of a validated in vitro model of traumatic brain injury.

## Results

### Mechanical scratch injury enhances HMGB1 release by microglia

**Figure 1:**
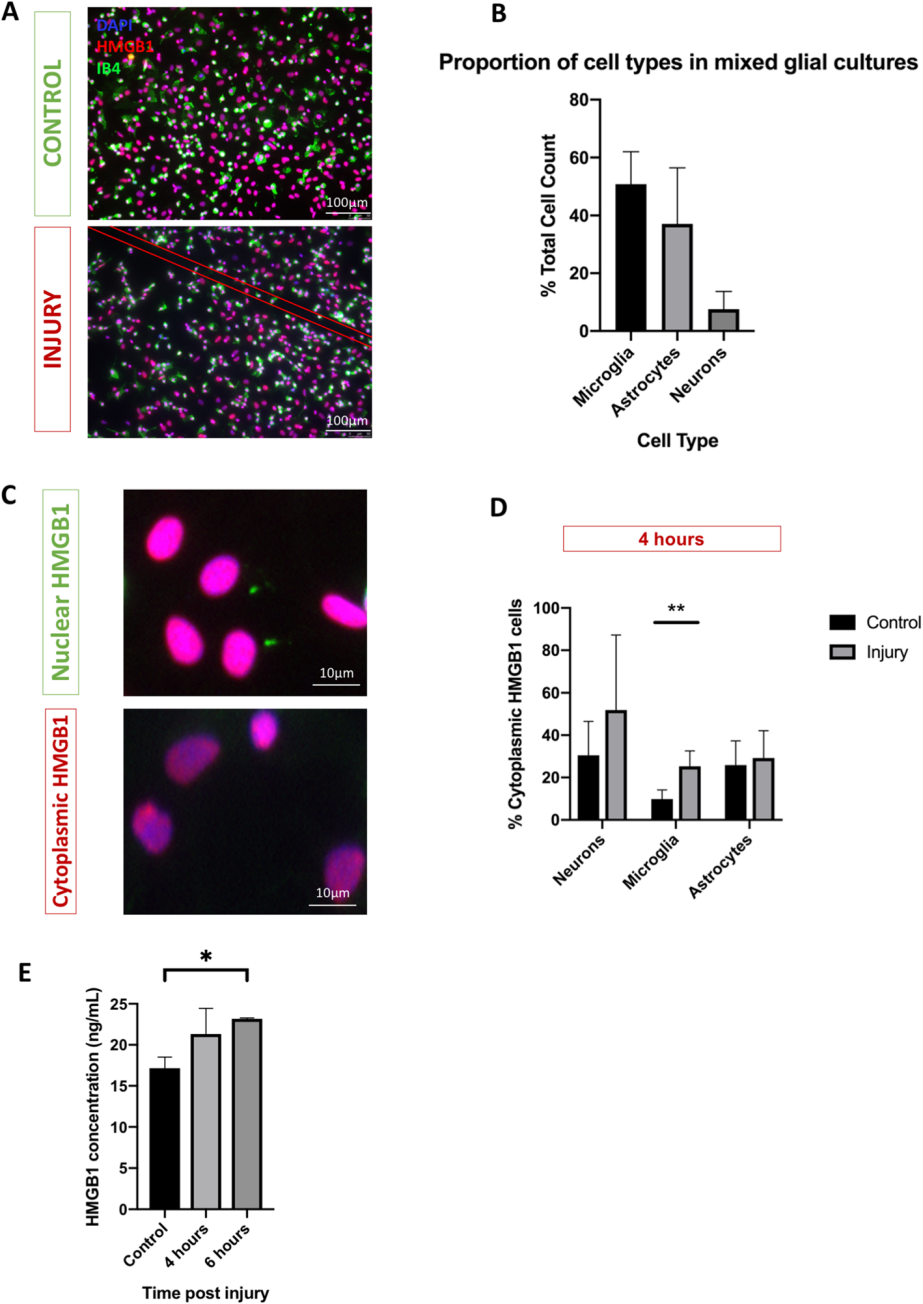
HMGB1 is released by microglia following injury. (**A**) Representative images of mixed neuro-glial cell cultures at 7 days in vitro: control (top panel) and scratch injury (bottom panel-site of injury indicated by red lines). (**B**) Mixed neuro-glial cell cultures consisted of microglia (IB4^+^ cells), astrocytes (GFAP^+^ cells), and neurons (Tuj1^+^ cells). (**C**) Representative images for nuclear (top panel) and cytoplasmic pattern (bottom panel) of HMGB1 staining (Red). (**D**) Demonstrates a hyperacute increase in the proportion of microglial (IB4^+^) cells with nuclear-cytoplasmic HMGB1 translocation at 4hrs post injury with relative to control. This did not reach statistical significance in Tuj1^+^ or GFAP^+^ cells. (**E**) ELISA assays revealed significant increases in extracellular HMGB1 concentration at 6 h post-injury. Data represent mean ± standard error based on a sample that represents at least 10 wells per condition from three different experiments. For comparisons between two conditions, two-tailed student’s T-test was used, and for multiple different conditions, a two‐way ANOVA and one-way ANOVA with Dunnett's multiple comparison test was used. p values of < 0.05 were considered significant (*p < 0.05; **p < 0.01; ***p < 0.001; ****p < 0.0001). cNSPCs = rat cortical neural stem cell progenitors; DIV = days in vitro.

HMGB1 activation is indicated by HMGB1 translocation from the nucleus to cytoplasm^19,20^. Mixed neuro-glial cell cultures were generated from rat cortex, consisting of microglia (IB4^+^ cells; 50.8 ± 11.2%), astrocytes (GFAP^+^ cells; 37.1 ± 19.4%), and neurons (Tuj1^+^ cells; 7.6 ± 6.1%) (Fig. 1B).

We subjected these cultures (7DIV) to needle scratch injury as described elsewhere^20–23^ and immunocytochemically quantified HMGB1 cellular sublocalisation at 4 h post-injury (Fig. 1A, C). We observed an overall increase in the proportion of cells with nuclear-cytoplasmic HMGB1 translocation (Fig. 1D). Examining the differential cell subtype contribution to HMGB1 activation, we found a significant increase in proportions of microglia (Ib4+) cells with cytoplasmic HMGB1 (25.3 ± 7.2 vs 9.8 ± 4.2% relative to control, *p* = 0.003). This did not reach statistical significance in neuronal (Tuj1 +) cells (51.9 ± 35.3 vs 30.5 ± 16.1% relative to control, *p* = 0.25) or astrocytes (GFAP + cells) (29.2 ± 12.9 vs 25.9 ± 11.4% relative to control, *p* = 0.67). We then examined whether injury induced HMGB1 nuclear to cytoplasmic translocation translates to extracellular HMGB1 release using ELISA essay. Our data demonstrated a statistically significant increase in HMGB1 concentration at 6 h post injury (17.1 ± 1.4 vs 23.2 ± 0.1 ng/mL; *p* = 0.02, Fig. 1E).

### Post-injury released HMGB1 acts via TLR2/4 receptors to reduce the numbers of oligodendrocyte progenitor cells in vitro

**Figure 2:**
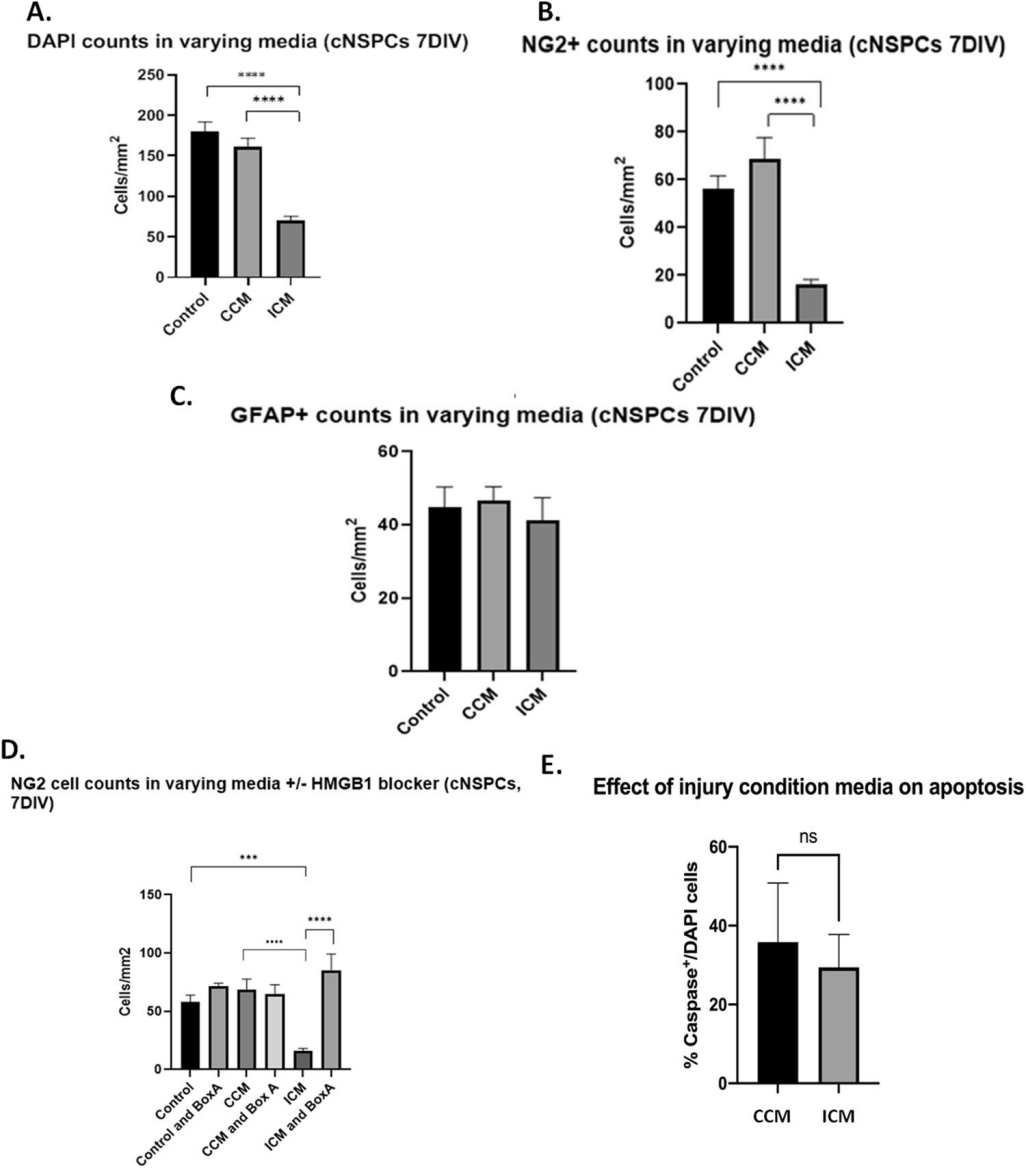
Post injury-released HMGB1 acts via TLR2/4 receptors to reduce the numbers of oligodendrocyte progenitor cells in vitro*.* (**A**) DAPI cell counts were significantly reduced in the presence of injury conditioned media, with no difference between control cultures and control conditioned medium. (**B**) NG2^+^ cells were significantly reduced in the presence of injury conditioned media compared to both control and control conditioned media. (**C**) There was no difference in GFAP^+^ cell counts in any of the conditioned media experiments. (**D**) Addition of BoxA, at a concentration of 100 ng/ml, did not affect NG2^+^ cell counts in control or control conditioned media conditions. However, BoxA rescued NG2^+^ cell counts in the presence of ICM to levels akin to the counts of control cultures (*p* < 0.0001). (**E**) Addition of ICM did not affect cell death in our cultures, assessed as the proportion of Caspase^+^ cells/DAPI^+^. For comparisons between two conditions, two-tailed student’s T-test was used, and for multiple different conditions, a two‐way ANOVA and one-way ANOVA with Dunnett's multiple comparison test was used. p values of < 0.05 were considered significant (*p < 0.05; **p < 0.01; ***p < 0.001; ****p < 0.0001). cNSPCs = rat cortical neural stem cell progenitors; CCM = controlled conditioned medium; ICM = injury conditioned medium; DIV = days in vitro.

Oligodendrocytes progenitor cells comprise most proliferating cells in the adult CNS; they play a key role in replacing injured oligodendrocytes and thus potential post injury axonal remyelination^24–26^. The exact mechanism to post-TBI OPCs loss is yet to be fully determined. In this set of experiments we examined the effect of injury condition medium (ICM) on the total numbers of cortical progenitor cells and the oligodendrocyte progenitor cells (NG2^+^ cells) in vitro. Treatment of cortical progenitor cells with control-conditioned medium (CCM) has no effect on the total (DAPI^+^) number of cells (179cells/mm^2^ ± 14.5 vs. 161cells/mm^2^ ± 10.5) (Fig. 2A). In contrast, treatment of cells with the ICM decreased the total number of cells from 179cells/mm^2^ ± 12.7 to 70cells/mm^2^ ± 5.4 (*p* < 0.0001) (Fig. 2A). Considering that NG2 cells constitute most of the dividing cells in the brain, we hypothesised that this dramatic drop in total number of cells is through detrimental effect on NG2^+^ cells. Indeed, treatment of cells with ICM resulted in a significant decrease in the numbers of NG2^+^ cells compared to standard control conditions (59cells/mm^2^ ± 5.8 SE vs.16cells/mm^2^ ± 1.9; *p* < 0.0001; Fig. 2B). To examine whether this HMGB1 detrimental effect is specific for NG2-expressiong cells or extend to involve astrocytes in cultures, we quantified the number of GFAP^+^ cells under the different experimental conditions outlined above. Analysis of astroglial cell subpopulations in the culture showed that 7 day exposure to ICM had no effect on the total numbers or proportions of GFAP^+^ cells (Fig. 2C).

To explore HMGB1 and its receptor mediation on the observed significant reduction in NG2^+^ cells, we co-treated cells in cultures with ICM in the presence or absence of BoxA, an antagonist of HMGB1 binding to TLR2 and 4 receptor subtypes^3,6^. The observed total cell counts and counts of NG2^+^ cells under control conditions and in control conditioned media were unaffected by the co-application of the TLR2/4 antagonist BoxA (Fig. 2D). Co-treatment with 100 ng/ml of BoxA did not affect NG2 cell counts in the control, (59cells/mm^2^ ± 5.7 vs. 71cells/mm2 ± 2.8; *p* = 0.511) or CCM (78.6 cells/mm^2^ ± 12.9 vs. 66cells/mm^2^ ± 7.9; *p* = 0.061) conditions, but completely abolished ICM-induced NG2 + cell loss (16cells/mm2 ± 1.8 SE vs. 85cells/mm^2^ ± 14.0; *p* < 0.0001; Fig. 2D). Application of ICM to cultures did not increase the proportion of Caspase^+^ cells compared to cultures exposed to CCM (Fig. 2E).

Taken together these observations implicate post mechanical injury release of HMGB1 has detrimental effects specifically on NG2^+^ cell counts, most likely through interactions with TLR2/4 receptors.

### Disulfide HMGB1 inhibits oligodendrocyte progenitor cells proliferation with no effect on the overall survival

**Figure 3:**
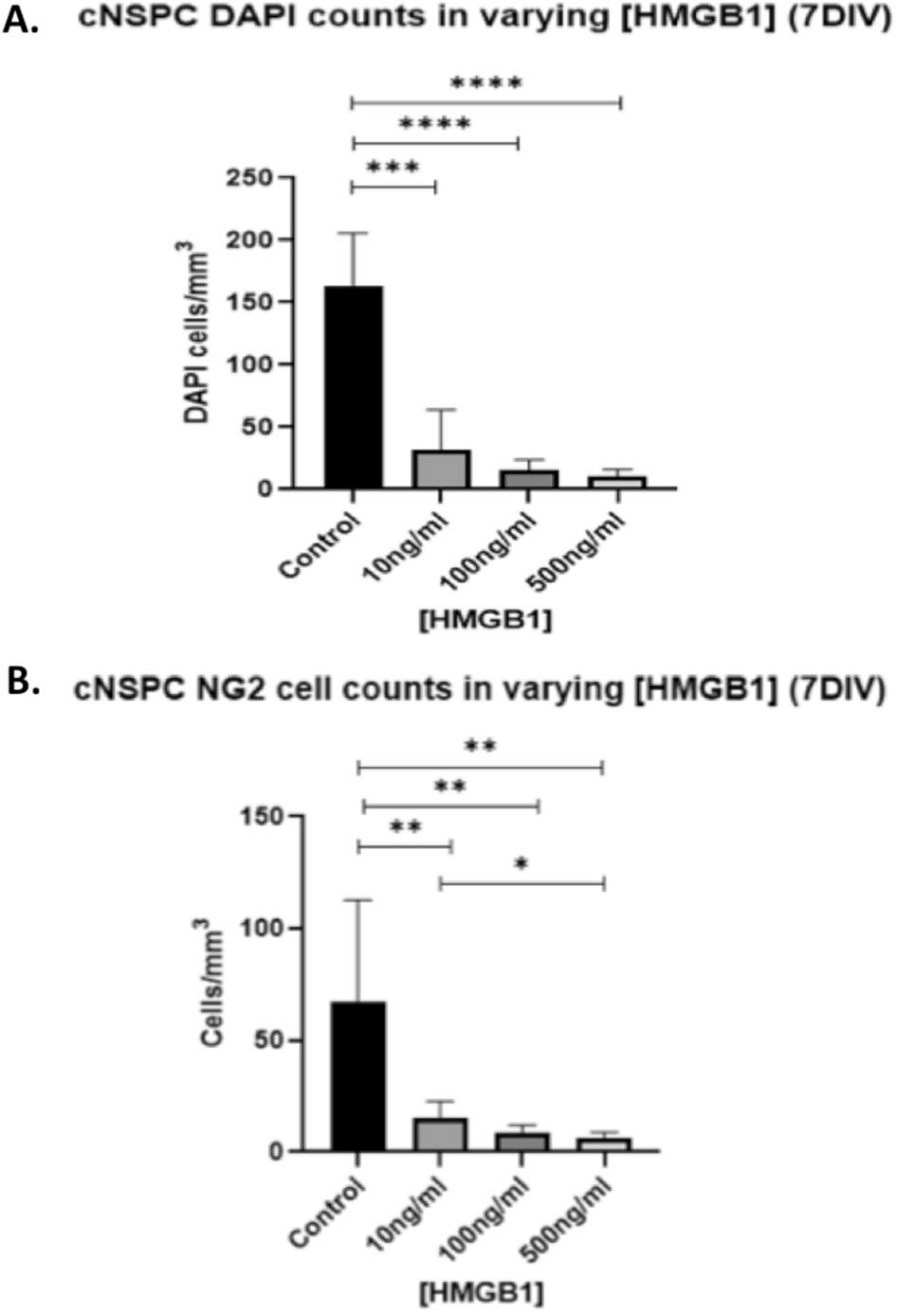
Disulfide HMGB1 reduces overall cell and NG2 + cell counts in vitro. (**A**) Addition of disulfide HMGB1 at concentrations of 10 ng/ml, 100 ng/ml and 500 ng/ml significantly reduced overall cell counts compared to control conditions. (**B**) Disulfide HMGB1 significantly reduced NG2^+^ cell counts in all experimental conditions, in a dose dependant manner. For multiple different conditions, a two‐way ANOVA and one-way ANOVA with Dunnett's multiple comparison test was used. p values of < 0.05 were considered significant (*p < 0.05; **p < 0.01; ***p < 0.001; ****p < 0.0001). cNSPCs = rat cortical neural stem cell progenitors; DIV = days in vitro.

Proliferation of OPCs is a key step in their immediate response to injury before they develop into mature myelin-producing oligodendrocytes^14,18^. 10–80 ng/ml of HMGB1 in the CSF and serum of patients sustained severe TBI is associated with worse clinical outcomes^2^. Three isoforms of HMGB1 have been identified based on the redox state: reduced, oxidised and disulfide HMGB1^6^. This last isoform has been consistently implicated in mediating HMGB1 inflammatory effects. We therefore set to examine the effects of the recombinant pro-inflammatory disulfide HMGB1 on OPCs proliferation and survival in neural stem cell progenitor cultures. In this set of experiments, cortical progenitor cell cultures containing NG2^+^ cells were exposed to increasing concentrations (10 ng/ml, 100 ng/ml and 500 ng/ml) of disulfide HMGB1 for 7 days DIV (IBL International). Treatment of cells in culture with as low as 10 nM HMGB1 resulted in a statistically significant reduction in the total numbers of cells (Fig. 3A) and in the total numbers of NG2^+^ cells (Control, 67cells/mm^2^; 10 ng/ml HMGB1, 15cells/mm^2^; 100 ng/ml HMGB1, 8cells/mm^2^; 500 ng/ml HMGB1, 6cells/mm^2^; Fig. 3B).

**Figure 4:**
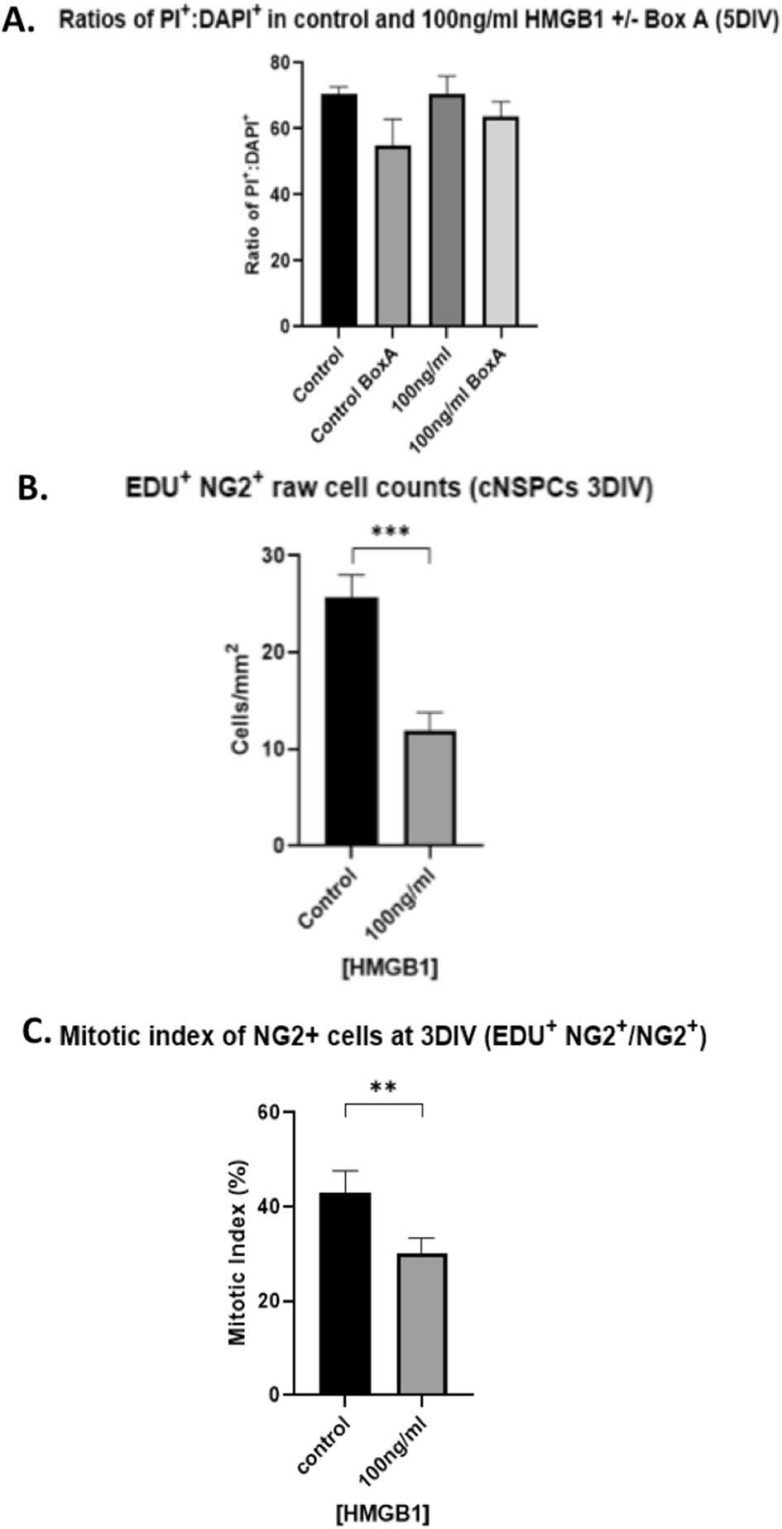
Disulfide HMGB1 inhibits oligodendrocyte progenitor cells proliferation with no effect on the overall survival. (**A**) Cell death was not significantly influenced by the presence of disulfide HMGB1. The addition of BoxA to cultures did not impact upon the proportion of PI + (dead) cells. Therefore, HMGB1, nor TLR4/2 blockade, affected overall cell death in these cell cultures. (**B**) NG2^+^ cell counts were significantly reduced in the presence of 100 ng/ml disulfide HMGB1 verses control cultures. (**C**) Disulfide HMGB1 at 100 ng/ml significantly reduced the mitotic index for NG2^+^ cells. For comparisons between two conditions, two-tailed student’s T-test was used, and for multiple different conditions, a two‐way ANOVA and one-way ANOVA with Dunnett's multiple comparison test was used. *p* values of < 0.05 were considered significant (*p < 0.05; **p < 0.01; ***p < 0.001; ****p < 0.0001). cNSPCs = rat cortical neural stem cell progenitors; DIV = days in vitro.

**Figure 5:**
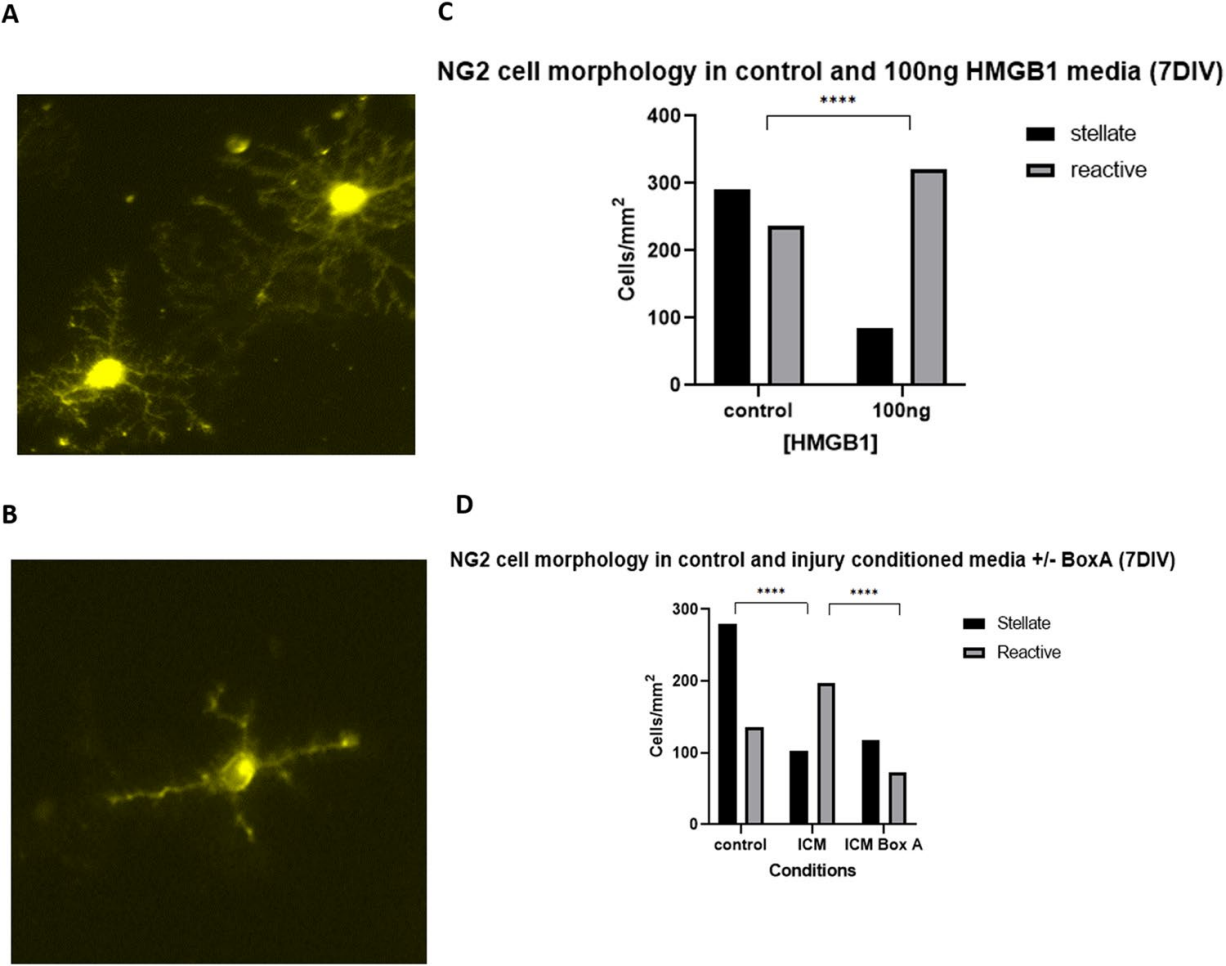
BoxA blocks ICM-induced shift in NG2 cells from stellate to reactive morphology. (**A**) Representative images of NG2^+^ cells in experimental cultures in a resting, stellate morphology. Note the radial pattern of numerous processes emerging from a homogenously stained cell body. (**B**) Representative images of NG2 cells in experimental cultures in a reactive morphology. Note the linear pattern of a much-reduced number (2–4) of processes emerging from a heterogeneously stained, swollen cell body. (**C**) In the presence of 100 ng/ml disulphide HMGB1, There were a significantly higher proportion of reactive cells (Chi-square 110.4, 1; *p* < 0.0001). (**D**) in the presence of injury conditioned media, there was again a significantly increased proportion of NG2^+^ cells with a reactive morphology (Chi-square 77.73; 1; p < 0.0001). Furthermore, addition of BoxA to ICM cultures reduced the proportion of reactive cells to that of control cultures (Chi-square 82.72, 2; *p* value < 0.0001). For comparisons between two different conditions, a two-sided Chi^2^ test was used in panels C-D. p values of < 0.05 were considered significant (*p < 0.05; **p < 0.01; ***p < 0.001; ****p < 0.0001). cNSPCs = rat cortical neural stem cell progenitors; DIV = days in vitro.

To examine whether HMGB1 enhanced overall cell death in cultures, cell death was assessed in live cultures using the death cell marker Propidium iodide (PI) as described previously^19,27^. We observed that 100 ng/ml of HMGB1 has no effect on the proportion of PI cells with respect to total number of cells (DAPI +) (70cells/mm^2^ ± 2.3 vs 70cells/mm^2^ ± 5.4, *P* = 0.32), indicating no anti-survival effect of HMGB1 in our cultures (Fig. 5). Furthermore, the addition of BoxA to the cultures had no influence upon cell death in (control: 70cells/mm^2^ ± 2.3 without BoxA vs 55cells/mm^2^ ± 8.1 with BoxA, *P* = 0.094; 100 ng/ml: 70cells/mm^2^ ± 5.4 without BoxA vs 64cells/mm^2^ ± 4.4 with BoxA, *P* = 0.36). This suggests that the TLR2/4 pathway did not affect cell survival (Fig. 4A).

Since cell death does not appear to explain the reduction in cell counts in our cultures, it is possible that HMGB1 may instead impact upon cell proliferation. To study this possible effect, cortical NSPCs were grown for 3DIV before being pulsed with the thymidine analogue EdU for the final eight hours prior to fixation. NG2^+^ cell counts were reduced in 100 ng/HMGB1 conditions, as per our previous findings (Fig. 4b) The mitotic index, (NG2^+^EdU^+^ cells/NG2^+^ cells) as a measure of the rate of NG2 + cell proliferation in the presence and absence of disulfide HMGB1, was quantified as described elsewhere^28^. 100 ng/ml of disulfide HMGB1 reduced the mitotic index of NG2 + cells to 30% (± 3.0) compared to that in 42% (± 4.4) in control conditions (*p* = 0.008, Fig. 4C). These observations demonstrate that disulfide HMGB1 isoform hinders OPCs proliferation at three days in vitro in our culture paradigm.

### BoxA blocks ICM-induced shift in NG2 cells from stellate to reactive morphology

NG2-expressing cells in the cerebral cortex respond to injury not only by increasing the rate of proliferation but also by changing their morphology and function^15^. In the developing and maturing cerebral cortex NG2^+^ cells temporally change their morphology in response to axonal myelination needs and can revert to basic morphology in response to injury^17,20^. We herein examined the effect of HMGB1 at 100 ng/ml and ICM on the morphology of NG2 cells. At 7 days in vitro we identified 2 different NG2 cell morphologies: (i) resting NG2 cells with multiple thin, branched processes extending radially from the cell body and (ii) reactive NG2 + cells with fewer, less branched, shorter processes and swollen cell bodies (Fig. 5A,B).

Our immunohistological analysis revealed a significant drop of the stellate, resting NG2 + cells from 290 cells under control conditions to 85 cells after exposure to 100 ng/ml of disulfide HMGB1. Consistently, HMGB1 treatment resulted in a significant increase of reactive NG2 cells (320 vs. 237 cells/mm^2^; (Chi-square 110.4, 1; *p* < 0.0001 Fig. 5C). Interestingly, ICM also resulted in a significant increase in reactive NG2 cells (102 vs. 197 cells/mm^2^; Chi-square 77.73; 1; *p* < 0.0001); BoxA treatment completely abolished this shift towards a reactive morphology (118 VS 72 cells/mm^2^ Chi-square 82.72, 2; *p* value < 0.0001; Fig. 5D). Taken together, disulfide HMGB1 and ICM shifted NG2^+^ cells from a resting stellate form to reactive phenotypes in these cultures; this effect was reversed by the blockade of TLR2/4 receptors using the HMGB1 antagonist BoxA.

## Discussion

Our study demonstrates that HMGB1 is released by injured cells into the extracellular environment (Fig. 1). When added to fresh cultures, both exogenously administered disulfide HMGB1, and ICM, influenced the number of NG2^+^ cells in our cultures (Fig. 2), possibly by influencing their rate of proliferation rather than cell survival (Fig. 4). Disulfide HMGB1 may therefore have an antiproliferative effect upon NG2^+^ cells at three days in vitro, which may explain its effect upon NG2^+^ cell counts at seven days in vitro identified in Fig. 3b. Furthermore, HMGB1 may also polarise NG2^+^  cells towards a reactive, activated phenotype as opposed to their resting state (Fig. 5). The impact of HMGB1 upon NG2^+^ cells exposed to mechanical scratch injury was also reversed when the TLR2/4 receptor pathway was blocked via the addition of BoxA to injury conditioned media, (Fig. 2) implicating this receptor pathway in the identified impact of disulfide HMGB1 upon NG2^+^ cell counts.

Identification of novel therapeutic options to improve patient outcomes after TBI has proved challenging. To this end, it is imperative that the pathophysiology and mechanistic understanding of the injury process undergoes further study. Recent years have seen a paradigm shift towards research directed towards understanding secondary brain injury, with identification of neuroinflammation as a key step in the brain injury process. However the repertoire of anti-inflammatory candidate agents is limited^6^ due to side effects and poor penetration of the blood–brain-barrier (BBB). In recent years, the DAMP HMGB1 has emerged as a potential novel anti-inflammatory target. Studies have identified that early targeting of HMGB1 via monoclonal antibodies, TLR2/4 blockade, and direct antagonism of HGMB1 via glycyrrhizin can reduce brain swelling, neuronal death and inflammatory cytokines following neurotrauma. Some of these agents are already in clinical use^9,29^ and thus refining our understanding of the HMGB1/TLR2/4 pathway in TBI is now a priority, as it is a promising therapeutic target. Furthermore, more attention has been directed towards white matter and demyelination injury following trauma, with greater appreciation of the importance of brain connections in normal brain function^17^. In models of mild demyelinating diseases, including TBI, remyelination has been demonstrated to take place through measurement of corpus callosum thickness and axon diameter^30^. However this process appears to be perturbed in more severe models and cases of neurotrauma^10,31^. Severe TBI is associated with poorer clinical outcomes^9^ recent neuroimaging studies have identified correlations with white matter disruption on diffusion tensor imaging following neurotrauma and the severity of TBI^32,33^. Analyses of the mechanisms of responses to TBI must therefore now consider the tissue specific damage to understand the potential for regional cell recovery and repair.

We therefore combined these avenues of research, i.e. assessment of the impact of HMGB1 as a neuroinflammatory modulator of TBI upon white matter oligodendrocyte progenitor cells, using an in vitro needle scratch injury model of TBI; we assessed how the pro-inflammatory disulfide isoform of HMGB1 may impact OPCs, and hence the potential for remyelination, following neurotrauma.

HMGB1 resides within the cell nucleus of in the resting state of neurones and glia^6^. We first set out to confirm whether HGMB1 is released by cells within our in vitro model of TBI. Our results identified that HMGB1 translocated to the cytoplasm, and was released into the extracellular milieu by neurones in cultures following mechanical scratch injury (Fig. 1). We utilised widefield fluorescence to score nuclear/cytoplasmic ratio, and ELISA to quantify HMGB1 presence in culture media as these methods are widely utilised an accepting in the existing TBI literature,^26,31,34–37^ The risk of confounding by variable expression and signal–noise ratio in our study is relatively small as we obtained high quality images with minimal background staining (Fig. 1C). Furthermore, whilst our ELISA data cannot confirm the cellular origin of HMGB1 in our cultures, our findings add support to previous studies which demonstrated HMGB1 release from microglia and injured neurones following traumatic injury models both in vitro and in vivo^33,34^. Furthermore, HMGB1 is significantly elevated in both serum and CSF following severe TBI in humans, to levels of between 100–500 ng/ml from baselines of < 10 ng/ml; these levels of systemic HMGB1 also correlated with clinical outcomes such as disability, identified through poor Glasgow Outcome Scale scores six months following brain injury^35,36,38^. Similarly to our in vitro findings, these elevations in HMGB1 occurred in the first 1–6 h following TBI, after which levels drop to baseline^35,38^. We therefore proceeded to compare control conditions with pulse of 100 ng/ml HMGB1 in our following experiments. Furthermore, HMGB1 released via necrotic neurones has been demonstrated to primarily be the disulfide isoform^6,39^, which is known to induce inflammatory responses in the CNS^40^. It is therefore likely that the HMGB1 present in the extracellular media (ICM) in our in vitro scratch model of TBI is predominantly the disulfide isoform.

Once HMGB1 is released into the extracellular settings following TBI, it binds to transmembrane receptors such as TLR2, TLR4, and RAGE^35^. Excessive inflammation resulting from activation of the HMGB1/TLR2/4 pathway in the brain has been implicated in TBI brain tissue injury, resulting in psychomotor deficits, cognitive issues, and epilepsy^6,35^. The impact of HMGB1 upon neurones has been studied, and the inflammatory cascades induced by this DAMP have been shown to result in worsening cerebral oedema, raised intracranial pressure, and neuronal death^3,6,33^.

However, it remains unclear how these inflammatory responses may influence white matter injury and repair following brain trauma, and whether modification of these processes may protect or aid regeneration of white matter following neurotrauma. The remainder of the present study therefore investigated how HMGB1 may impact upon oligodendrocyte precursor cells in vitro, and hence how it may influence white matter injury and repair following TBI. We also defined the potential receptor mediation of these effects via experimental blockade of TLR 2/4 in culture.

Demyelination is known to occur following TBI^10^. Remyelination therefore presents an opportunity for plasticity and recovery of function after neurotrauma. A Remyelination of injured axons is dependent upon the actions of Oligodendrocyte precursor cells, (OPCs). These cells can be identified through their expression of cell surface markers A2B5, PDGFR and NG2. These NG2^+^ cells (OPCs) constitute a significant pool of dividing cells in the brain, and are known to be able mature into myelinating oligodendrocytes. Models of multiple sclerosis have identified that OPCs proliferate and migrate to sites of inflammatory injury, where they either a) mature and contribute to remyelination and recovery of axon function or b) fail to proliferate and mature into myelinating oligodendrocytes, which leads to worsening cell death and/or white matter recovery^14^. We identified that post-mechanical injury release of HMGB1 had detrimental effects on NG2^+^ cell counts in our cultures when compared to control media, and control conditioned media (Fig. 2B). Since NG2^+^ cell counts were rescued upon inclusion of BoxA to cultures, this effect is most likely through interactions with TLR2/4 receptors (Fig. 2D). This implies that the ICM either potentiates NG2^+^ cell death, reduces NG2^+^ cell proliferation, or both. Since we identified that cell death was not significantly increased in our scratch injury model, (Fig. 2E) the effect might more likely be anti-proliferative rather than anti-survival in these cultures. This implicates HMGB1, which we demonstrated is released into the ICM used in this TBI model, (Fig. 1E) as a modulator or OPC survival and/or proliferation in culture. Interestingly, this effect was not seen upon astrocytes, (Fig. 2C) suggesting that HMGB1 may be a specific modulator of OPC survival and/or proliferation, and hence white matter repair, following neurotrauma. Future in vivo studies could incorporate HMGB1^−/−^ models to provide evidence that these effects are medicated via HMGB1-signalling. Notwithstanding this need for further data, other published in vivo work involving HMGB1-receptor knock-out mice provides some evidence which corroborates our results, with TLR4^−/−^ mice demonstrating reduced oligodendrocyte loss following injury^2^. However some studies in spinal cord injury have suggested that TLTR4^−/−^ mice exhibit more severe motor deficits and demyelination compared to wild type^37,41^. TLR4 may thus exhibit protective effects under certain conditions. These may relate to the presence of other ligands for TLR4, changes in the isoform of HMGB1 being secreted, (disulfide Vs redox isoforms) and differences in white matter lesion severity^2,6,17^.

HMGB1 activation of RAGE, TLR2, and TLR4 leads to the phosphorylation of several mitogen-activated protein kinases (MAPKs) that activate the downstream transcription factor nuclear factor kappa-light-chain-enhancer of activated B cells (NF-κB) and generates an inflammatory cell response^2,3^. These signaling cascades may also contain elements amenable to therapeutic intervention to dampen damaging neuroinflammation post-TBI. However, such agents often have significant side effects and/or may not easily penetrate the BBB to exert their protective effect. HMGB1 may therefore represent a therapeutically desirable ‘master switch^2,42^ of neuroinflammation, modulation of which may be able to control deleterious neuroinflammatory responses after TBI.

However, some work has implicated HMGB1 as a promotor of recovery following brain injury. Mature oligodendrocytes exposed to ICM containing HMGB1 were more resistant to hypoxic insults than control oligodendrocytes^11^. Furthermore, in vivo models of stroke demonstrate that HMGB1 blockade worsened sensorimotor function in rodents^43^. These results contrast to our findings of HMGB1 being detrimental to white matter tissue but may be related to differences in the isoform of HMGB1 released after ischaemia Vs neurotrauma; these studies utilised ICM derived from oxygen deprived oligodendrocytes, rather than neuronal and mixed glial cell release of HMGB1 after mechanical injury. It is thus possible that the ischaemic models which identified protective effects of HMGB1 involved release of oxidized HMGB1 rather than disulfide HMGB1, the former of which is thought to have anti-inflammatory actions^44,45^. Furthermore, the receptor mediation of these effects on mature oligodendrocytes may differ to that upon OPCs^11^.

We identified a dose–response relationship between disulfide HMGB1 concentration and NG2^+^ cell counts in culture (Fig. 3B). We conducted cell death experiments as described by our research group and others previously^46,47^, to further assess this effect. These experiments did not identify any change in cell death in our cultures (Fig. 4A). However, proliferation assays using EdU identified that HMGB1 exposure reduced the mitotic index of NG2 + cells at 3 DIV in our cultures (Fig. 4C). This may explain the underlying the impact HMGB1 had on total NG2^+^ cell counts after 7 DIV in our culture paradigms. There is some evidence that brain injury can increase proliferation of OPCs in the region of injury^17,48–51^. However, these models primarily assess NG2^+^ cell proliferation 1–6 weeks post-injury, whilst our work assessed proliferation in the immediate hours following exposure to HMGB1. Indeed, reduced proliferation of NG2^+^ cells in the first days following stab injury to the brain has also been reported by others^17^. Therefore, our results may reflect the acute impacts disulfide HMGB1 release from injured neurones can have upon OPC proliferation. Furthermore, electron microscopic examination of traumatic CNS lesions has identified that NG2^+^ cells at the epicentre of lesions are significantly reduced and do not appear to proliferate, in contrast to NG2^+^ cells at the periphery of lesions^25,26,36^. Our experiments included exposure to pathological concentrations (100–500 ng/ml) of HMGB1, which may represent the concentrations of HMGB1 at the focus of traumatic lesions, where OPCs proliferation might be reduced when compared to the overall NG2^+^ cell proliferation at the lesion edges, where HMGB1 concentrations may be significantly different to the epicentre^25,48–50^.

We identified that HMGB1 impaired OPC proliferation in the early stages post-exposure, and others have established that HMGB1 concentrations in the initial phase post-TBI correlate with poor clinical outcomes^33^. Therefore, higher exposure to disulfide HMGB1 at the time of injury may affect the ability of OPCs to proliferate and therefore impair the potential for later remyelination of injured axons. Further analysis of OPC proliferation at further time points in our model may help to identify whether the rate of proliferation of OPCs changes following exposure to clinically relevant concentrations of disulfide HMGB1.

Our study also identified two broad NG2^+^ cell morphologies within our cultures (Fig. 5A, B). Stellate NG2^+^ cells have been described as the default, resting state, which should constitute the majority of NG2^+^ cells in healthy brain tissue^15,24,48^. The second, reactive morphology is associated with states of neuroinflammation and neurodegeneration, with up to 50–100% of NG2^+^ cells displaying this morphology in neuroinflammatory pathologies^15,20,24,52^. In our experiments, application of pathological concentrations of HMGB1, and of ICM, shifted the predominant NG2^+^ cell morphology to the reactive state (Fig. 5C, D). This impact upon NG2^+^ cell appearance was abolished by addition of BoxA, implicating TLR2/4 as a mediator of this change in cell morphology.

The reactive NG2^+^ cell morphology includes reduction in the arborisation and number of processes increased soma granularity, and large, oval-shaped nucleus^15,20,24,53^. These cells are also implicated as major contributors to the ‘glial scar’ which develops after severe TBI; a collection of glial cells which impair remyelination and functional recovery after trauma^24^. Our cultures demonstrate increased reactive NG2^+^ cells in HMGB1 exposed cultures at 7 DIV, (Fig. 5) by which time when many of the reactive OPCs would have been expected to begin to mature into myelinating oligodendrocytes^53^. The persistence of an increased proportion of reactive NG2^+^ cells after exposure to pathological levels of HMGB1 in our cultures may thus present OPCs which began, but failed to proceed with, appropriate proliferation and maturation into myelinating oligodendrocytes^26^. This may be due to their exposure to disulfide HMGB1 and its activity through TLR2/4. Formal quantification of NG2^+^ cell morphology in these cultures could be performed using Scholl analysis^20^. However, in our cultures the features of reactive NG2^+^ cells were easily identifiable, with only 1–2 sparsely branched processes per cell compared to multiple, significantly arborised dendrites defining the resting cells (Fig. 5A, B). We were therefore felt able to confidently categorize NG2^+^ cells as reactive or resting based on immunocytochemical appearances.

It is important to acknowledge that the morphological changes we identified have been described as markers of oligodendroglial differentiation and maturation^54,55^. However, when OPCs mature in development they should stop expressing NG2 and begin expressing markers of more mature oligodendrocytes, such as Olig 2, O4 and eventually MBP^56^. OPCs in our cultures were NG2^+^, and therefore were not in a mature or differentiated state yet. As such, whilst their morphology may resemble that of differentiated oligodendroglial cells, the ongoing expression of NG2 suggests that differentiation was not the cause of the identified morphologies; this notion is in line with more recent studies which have confirmed that oligodendroglial precursors adopt the morphologies described (resting Vs reactive) in response to environmental cues^15,20,24,57–59^. To formally confirm the implications from this section of our data, assessment for markers of oligodendroglial differentiation alongside morphological analysis will be a key step for the future.

Whilst NG2^+^ cells are often synonymous with OPCs, other cells can express NG2^+^ following ischaemic or toxic insults, such as astrocytes^28,60^. It may be desirable to confirm the identify of NG2^+^ cells in our cultures as true OPCs through dual labelling with PDGF and/or A2B2^3,6,61^. However, we did not identify any cross-reactivity in our cultures between GFAP^+^ nor TUJ^+^ and NG2^+^ cells, (data not shown) so it is unlikely that our NG2^+^ cell counts are significantly biased by NG2^+^ expression by other cells.

In addition to TLR 2/4, disulfide HMGB1 also activates the Receptor for Advanced Glycation End Products, (RAGE^3,6,61^) and assessment of how this receptor pathway influences OPCs warrants investigation. So far, the HMGB1-RAGE pathway has been implicated to induce endothelial progenitor cell proliferation in vitro when assessing animal models of stroke^62^. Whether this effect is detrimental, or enhances recovery of white matter cells after injury, remains to be determined^2^.

To accurately model the acute release of HMGB1 following brain trauma^33^, our study paradigms incorporated an acute pulse of HMGB1 or ICM, followed by cell culture and subsequent immunohistochemical analysis. Whilst this mirrors the in vivo finding that HMGB1 rises acutely following injury, and then rapidly falls to baseline levels, it may not mirror the concentrations of HMGB1 at the site of injury over time. The temporal changes in HMGB1 concentration at the local site of brain injury, and indeed potential changes in the isoform of HMGB1, remain to be defined.

We utilised mixed cultures of neurones and glia to reflect the mixed cell populations in the brain cortex in vivo. It is therefore possible that the impacts we report of HMGB1 upon NG2^+^ cells may be mediated via other cells present in our cultures, such as astrocytes or microglia. Pure OPC cultures, with and without neuronal co-culture are feasible^53^ and would help to define whether the effects of disulfide HMGB1 upon OPCs in our model are direct or indirect. Furthermore, it may facilitate deeper assessment of the impact disulfide HMGB1 may have upon OPC maturation and myelination. It is known that OPCs need to migrate to sites of injury, and appropriately mature into oligodendrocytes to be able to remyelinate axons following neurotrauma^6,10^. Failure of these processes may underly the formation of ‘glial scar’ tissue, which represented clusters of numerous glia which are detrimental to axonal recovery post-TBI^2,51^. Disulfide HMGB1 may thus impact upon OPC maturation and subsequent oligodendrocyte myelination capacity, as well as OPC proliferation, morphology, and cell numbers. Kerman et al. describe experiments wherein OPCs can be matured in culture to oligodendrocytes, and then cultured alongside neurones to assess their ability to myelinate axons^63^. Such a paradigm could be incorporated into experiments exposing OPCs to disulfide HMGB1 to further assessment of how this DAMP may influence remyelination following models of brain trauma.

It remains feasible that the effects we have presented may be due to direct HMGB1-signalled to OPCs, or represent paracrine signalling to other cell types in our cultures which then modulate NG2 cell proliferation. It has been demonstrated that OPCs express the HMGB1 receptor TLR2,^64,65^ that microglia and neurones express both TLR2 and 4 in vitro^56,66–68^*,* and modulation of TLR signalling can influence oligodendroglial cells in the spinal cord and brain^65–68^. Assessment of the impacts of HMGB1-signalling in pure OPC cultures, and in myelinating co-cultures, is required to further elucidate whether the impact of HMGB1 upon white matter precursors is a direct effect, or whether it is mediated via HMGB1-TLR signalling on other cell types in vitro.

## Conclusion

Identification of neuronal, microglia and astrocyte secretion of HMGB1 following severe TBI in humans, and OPC-specific toxicity of HMGB1 in vitro*,* suggests that HMGB1 may be a potential therapeutic target for improving morbidity associated with TAI. Higher exposure to disulfide HMGB1 may impact upon OPC proliferation following injury, negatively affecting the potential for recovery of the mature oligodendrocyte population, and thus subsequent remyelination of axons. This may in part be mediated via a TLR2/4 dependant mechanism.

## Methods

All animal experiments and procedures were conducted within the remit of the UK Animals (Scientific Procedures) Act 1986, and all methods were approved by local ethical review by the Cardiff University research ethics committee, and relevant personal project licence was in place (P8157151A) as outlined by Cardiff University School of Psychology. All attempts were made to ensure the comfort and respect of the animals during the study. The animals were kept in a controlled environment maintained with a 12-h light/dark cycle. Rats had access to food and water ad libitum.

### Animal cortical tissue monolayer culture

Cortical neural stem/progenitor cells (NSPCs) generated from postnatal (P8-10) *Sprague Dawley* rats as described elsewhere^46^ and cultured in serum free media. Briefly, using sterile conditions, animal cortices were dissected and cut on a MacIlwain tissue chopper into 40 µm-thick slices in Gey’s balanced salt solution (Life Technologies, Paisley, UK) supplemented with 4.5 mg/ml glucose at 4 °C. The tissue slices were then digested with 2 mg/ml papain (22.0 U/mg, Sigma) in pre-warmed Neurobasal A (NB), supplemented with 2% B27 (Life Technologies) and 0.5 mM Glutamine (Sigma, UK) for 30 min at 37 °C. After washing, cell release was achieved by trituration for about 10–15 times with Pasteur pipette in NB/B27 Glutamine medium. NSPCs were purified free of debris and enriched on a 2-step density OptiPrep (Axa-shields, Oslo, Norway) gradient. Viable cells were then seeded at a density of 100,000 viable cells per ml in NB/B27 and Glutamine directly onto poly-l-lysine coated glass cover slips in 24-well plates. Cells were incubated under (5%CO2/95% air at 37 °C) incubator conditions. At 2 h after plating the medium was replaced by fresh medium with or without the HMGB1 (100 ng/ml). All media included a combined antibiotic/antimycotic (Penicillin/ Streptomycin and Fungizone, Life Tech, USA). For cultures longer than three days, two thirds of the culture media were replaced every 3 days. For cultures investigating the effects of HMGB1 blockade, Box A, a protein fragment of HMGB1 which competes with endogenous HMGB1 for binding sites on Toll-like receptors 2 and 4, without activating the receptor’s intracellular domain^10^. Box A can therefore attenuate the biologic function of the full-length HMGB1, thus can be utilised experimentally as a specific antagonist of HMGB1 effects via TLR2 and TLR 4. Box A was added to relevant cultures at the point of cell plating, at a concentration of 30 ng/ml.

### Assessment of cell death

Cell death in live cultures was quantified using the cell death marker Propidium Iodide (PI) and the nuclear stain DAPI as described previously^46^. PI was added to cultures of living cells at 5 µg/ml for 40 min at 37 °C. Cells were then incubated in NB/B27 and Glutamine containing DAPI (20 µg/ml) for another 40 min. Finally, DAPI containing medium was removed and cells were maintained in fresh culture medium (NB/B27 and Glutamine) while imaged (6 systematic randomised fields per well). The proportions of non-viable cells (PI stained) of the total (DAPI stained) cell population were then calculated. Cell death was also corroborated with immunofluourence detection of the ratio of Caspase, a marker of apoptosis.

### Immunohistochemistry

Immunofluorescent staining was performed on 4% paraformaldehyde (PFA)-fixed cells as described in detail by our research group^46^. The following primary antibodies were used in PBS-0.1% Triton-X100: mouse anti-rat NG2 1:500 (Pharmingen), goat anti–GFAP 1:500 (Sigma), rabbit anti-TuJ1 1:500 (Millipore). Following three PBS washes, primary antibodies were probed by Cy2 anti-rat (1:500) and anti-mouse and anti-rabbit (1:500) in PBS-Triton 0.1% for two hours at room temperature.

Cells were then counterstained with the nuclear stain 4′-6-diamidino-2-phenylindole (DAPI) (5 µg/mL, Sigma), and total cell count was determined by determining the number of DAPI positive cells. Staining was visualised using fluorescent light microscopy (Improvision, Leica Microsystems UK Ltd). Primary antibody negative controls showed no staining.

Morphological analysis of NG2^+^ cells was performed as described previously^15,24^, via visual analysis of immunocytochemical appearances. The activity state of NG2^+^ cells was estimated based on previously described changes in NG2^+^ cell morphology^54,59^. In brief, a stellate appearance with numerous (> 5) radial processes signified NG2 cells in the resting state, verses swollen cell bodies, with much reduced numbers (1–4) of processes and a non-radial morphology signifying reactive NG2 cells^15,24,59^. Quantification of HMGB1 nuclear-to-cytoplasmic translocation was accomplished as described by our research group elsewhere^54^.

### ELISA experiments

The levels of HMGB1 were measured in condition medium under control conditions and at 4 and 6 h post injury using an ELISA kit (from R&D systems and IBL International respectively) according to the manufacturer protocol. After preparing the calibrators (starting from the stock solution to produce a twofold dilution series), quality controls, and samples, the plate was loaded. The first two lines of the plate were loaded with calibrators with the following concentrations: 2000, 1000, 500, 250, 125, 62.5, and 31.2 pg/ml to reproduce a standard curve. The other lines were loaded with controls and the samples (injury condition medium), each to a final volume of 100 µL per well. The plate was incubated for 2 h at room temperature. After that, each well was aspirated and washed (with a wash buffer), repeating the process four times for a total of five washes. After the last wash, any remaining wash buffer was removed by aspirating and inverting the plate and blotting it against clean paper towels. A 100 µL of human HMGB1 Conjugate was added to each well and the plate was then incubated for 2 h at room temperature. After a new aspiration/wash step as described before, 100 µL of Substrate Solution was added to each well and incubated for 30 min at room temperature (Protect from light). Finally 100 µL of Stop Solution was added to each well and determined the optical density of each well within 30 min, using a microplate reader set to 450 nm. The absorbance of the resulting colour change was measured spectrophotometrically and calculated as proportional to that of the HMGB1 calibrators’ concentrations.

### Assessment of cell proliferation

EDU detection was carried out using established Click-iT protocol (Thermofisher)^69^. After three days in vitro, EdU was added directly to NSPCs for the final eight hours at a concentration of 10 μM, before cells were rinsed once in PBS and fixed in 4% paraformaldehyde (PFA) for 30 min at 4 °C. We used the proportion of NG2^+^ cells that entered the S phase (EDU^+^) with respect to total number of NG^+^ cells to calculate the mitotic index of these cells under different conditions.

### In vitro scratch model of TBI

We used a validated in vitro model of TBI to further assess the impact of HMGB1 and its blockade upon cortical NG2 cells^22,23^. Sprague–Dawley rat cortical mixed glial cells were cultured as per the protocol outlined previously^22,46^. After six days in vitro, half of cell culture wells underwent controlled scratches in orthogonal directions across the plates, with the other half undergoing a routine media change. After a further twenty-four hours in vitro, the media from these cultures was collected and applied to cortical NSPCs after six days in vitro. After a 24-h pulse with either control or injury conditioned media, (CCM and ICM respectively) with or without the presence of Box A, NSPCs were fixed and immunostained as described above.

### Imaging and data analysis

All experiments underwent a minimum of three repeats and the researcher performing cell counts was blinded to each condition. Cell counts were validated as consistent by two independent researchers. Imaging of cells in culture was performed on a Leica DM6000B Upright System microscope (Leica, Germany). The area of a 20 × field was measured using a 255 µm grid graticule slide (Microbrightfield, Williston, USA). Cell counting was performed on six random fields per well using ImageJ software^70^. Raw data from the field counts were averaged and plotted ± SEM and expressed as cells/mm^2^ per well, based on a sample of four to eight wells per condition per experiment. All experiments were repeated at least three times. One experiment consisted of two-four cortices from two-six animals, pooled and prepared as described above. Graphpad Prism software was used for statistical analysis (GraphPad, Inc., San Diego, CA, http://www.graphpad.com). Statistical significance was assessed with either Students’ t test for single comparisons or with one and two way ANOVA, followed by appropriate post hoc tests (see Figure legends) for multiple comparisons (*p* < 0.05 considered significant).
